# Neuroblastoma accompanied by hyperaldosteronism

**DOI:** 10.12861/jrip.2014.23

**Published:** 2014-07-01

**Authors:** Kaan Gulleroglu, Umut Bayrakci, Sibel Tulgar Kinik, Nihal Uslu, Alev Ok Atilgan, Faik Sarialioglu, Esra Baskin

**Affiliations:** ^1^Department of Pediatric Nephrology, Baskent University, Ankara, Turkey; ^2^Department of Pediatric Endocrinology, Baskent University, Ankara, Turkey; ^3^Department of Radiology, Baskent University, Ankara, Turkey; ^4^Department of Pathology, Baskent University, Ankara, Turkey; ^5^Department of Pediatric Oncology, Baskent University, Ankara, Turkey

**Keywords:** Neuroblastoma, Hypertension, Hyperaldosteronism

## Abstract

**Background:** Tumors known derived from kidneys which take place in secondary hyperaldosteronism etiology are juxtaglomerular cell tumor and Wilms’ tumor. Neuroblastoma presenting with hyperaldosteronism is rare.

**Case:** A 15-month-old girl who had been having diarrhea and fever for 2 weeks presented with a 3 day history of bilious vomiting, metabolic acidosis and severe hypokalemia. She was referred to our hospital with the pre-diagnosis of unknown manifest hypertension etiology, diarrhea, and paralytic ileus after having therapy-resistant hypokalemia and severe resistant acidosis. On her examination after being admitted to our clinic, she was weak, unwell and lethargic with a blood pressure of 140/93 mmHg. Due to the hypertension and severe hypokalemia, the patient was considered to be hyperaldosteronism. Serum aldosterone level, plasma renin activity and cortisol level were elevated. Radiologic findings were compatible with neuroblastoma. The patient underwent an abdominal surgery and the mass excision. The histopathological examination was proved neuroblastoma.

**Conclusion:** Hyperaldosteronism can be presented by unexpected atypical forms as in our patient

**Implication for health policy/practice/research/medical education:**

Physicians should be aware that hyperaldosteronism can be presented by unexpected atypical forms as in our patient. To sum up, imaging techniques and pathological diagnosis have to be considered to determine the etiology of hyperaldosteronism.

## Introduction

Endocrine causes of hypertension in childhood are rare, but usually treatable and often curable (1). Hypertension with hypokalemia and suppression of plasma renin activity is known as mineralocorticoid hypertension. The most common cause of mineralocorticoid hypertension is probably primary aldosteronism. Three monogenic forms of mineralocorticoid hypertension have been described: glucocorticoid-suppressible hyperaldosteronism, Liddle’s syndrome, and apparent mineralocorticoid excess, which have provided new insights into mineralocorticoid hormone action (2). Hyperaldosteronism is a group of disease which is characterized by hypertension, hypokalemia, alkalosis, urinary potassium excretion and increased levels of both urinary and plasma aldosterone. Tumors known to be secreting renin and derived from kidneys which take place in secondary hyperaldosteronism etiology are juxtaglomerular cell tumor, Wilms’ tumor, renal adenocarcinomas, renal oncocytomas and cortical adenomas (1). Neuroblastoma presenting with hyperaldosteronism is rare. We report a case of neuroblastoma presenting with hyperaldosteronism.

## Case presentation

**Figure 1:**
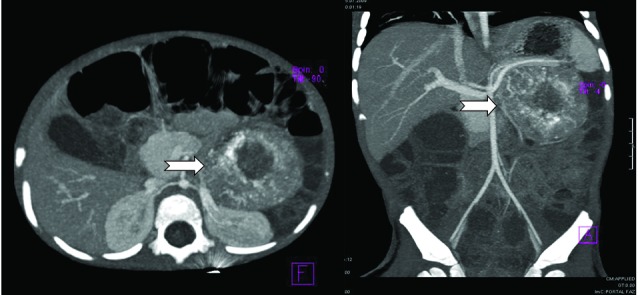
Abdominal-CT findings were compatible with neuroblastoma.

**Figure 2:**
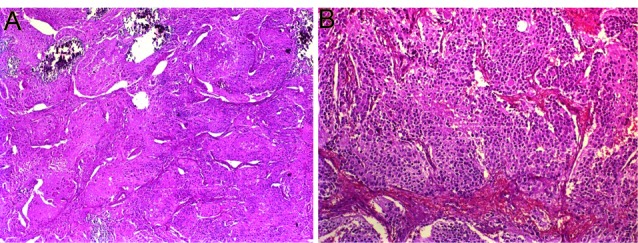
A) Differentiated neuroblastic and ganglion cells on calcified stroma, H&E, x40. B) Differentiated neuroblastic and ganglion cells, H&E, x100.

A 15-month-old girl who had been having diarrhea 10-15 times per day, intermittent vomiting and fever for 2 weeks presented with a 3 day history of bilious vomiting, metabolic acidosis and severe hypokalemia (K: 1,6 mEq/L). She was treated at another hospital for gastroenteritis and paralytic ileus, and was referred to our hospital with the prediagnosis of unknown hypertension etiology, acute gastroenteritis and paralytic ileus after having therapy-resistant hypokalemia, acidosis, diarrhea and manifest hypertension. On her examination, after being admitted to our clinic, she was weak, unwell and lethargic with a blood pressure of 140/93 mmHg. She had severe dehydration and hyperactive bowel sounds, while other systemic examination was found normal. Abdominal ultrasound scan was normal. Laboratory investigations showed metabolic acidosis (arterial blood pH: 7.32, HCO3: 7.2 mmol/L), hypokalemia (K: 1.6 mEq/L), hyponatraemia (130 mEq/L), and leukocytosis (15900/µL). Hemoglobin level was 12.7 g/dL along with polymorphonuclear leukocytes predominant peripheral blood film and platelet count of 445.000/µL. Further blood tests evaluating renal functions showed that BUN was 18 mg/dL, serum creatinine was 0.89 mg/dL. Also serum total calcium and phosphorus were 10 mg/dL and 2.24 mg/dL respectively. Although she had been suffering from therapy-resistant diarrhea and severe dehydration for a long time. Daily urine output was 3.2 ml/kg/hr. Urinanalysis results included urine specific gravity of 1006, a pH of 6, and protein of 150 mg/dL. Direct urine microscopy was normal. The urine spots protein: creatinine ratio was 4.32. Albumin was found 4.95 g/dL. Fractional excretion of sodium was 1.4%. Fractional excretion of potassium was 7.2%. Serum anion gap was assessed as 25.8 mEq/L while the urine was -7.1 mEq/L. Due to the hypertension and severe hypokalemia, the patient was considered to be hyperaldosteronism and some tests are conducted. Serum aldosterone level was found 1200 pg/mL (70-540), plasma renin activity 2.77 ng/mL/s (0.5-2.6 ng/mL/s) and cortisol level 50 mcg/dL (5-25 mcg/dL). While the two previous abdominal ultrasound scans were found to be normal, however, on physical examination of abdominal a suspicious mass was found. Thus, a repeat abdominal ultrasound scan was performed for especially evaluating the adrenal glands. Ultrasound showed a heterogeneous, vascular, solid left adrenal mass measuring 6×5 cm which contains calcification. Abdominal-CT findings were similar to ultrasound and to be compatible with neuroblastoma. Urine levels of vanilmandelic acid (VMA) and serum neuron specific enolase (NSE) levels were elevated which were evocative of a neuroblastoma. The patient underwent an abdominal surgery and the mass excision was performed by pediatric surgeons. Following the tumor excision, therapy-resistant hypertension became normal, diarrhea ceased and the need for fluid and electrolytes replacement caused by the severe diarrhea ended. The histopathological examination revealed a tumor measuring 6×5×4.5 cm with differentiated cells containing large necrosis and calcification suggestive of a neuroblastoma. The surgical margins were free of tumoral invasion. The patient was evaluated as low risk according to the TPOG-2009 protocol, discharged from the hospital and recruited for follow up visits (Figures 1 and 2).

## Discussion

Hyperaldosteronism is a group of disease which is characterized by hypertension, hypokalemia, alkalosis, urinary potassium excretion and increased levels of both urinary and plasma aldosterone. The adrenal gland is formed of two different parts which are originated from two different embryonic origins; adrenal medulla and adrenal cortex. Aldosterone is a steroid hormone produced by zona glomerulosa (outer section) of the adrenal cortex (3). Aldosterone secreting adenomas, adrenal hyperplasia and other adrenal abnormalities are considered to be the causes of primary hyperaldosteronism. A number of conditions associated with hyperreninemia result in secondary hyperaldosteronism. Tumors known to be secreting renin and derived from kidneys which take place in secondary hyperaldosteronism etiology are juxtaglomerular cell tumor, Wilms’ tumor, renal adenocarcinomas, renal oncocytomas and cortical adenomas. Severely increased plasma renin activity is seen especially in juxtaglomerular cell tumors among all while the others have fewer renin secreting cells and due to this, less high plasma renin activities (4). Neuroblastoma which arises from the adrenal medulla differs from those groups of tumors which are mentioned above. Neuroblastoma presenting with hyperaldosteronism is rare. This effect of neuroblastoma can be explained by increased renin secretion due to the mass effect of tumor pressing on the renal artery (5). Enterohormones (vasoactive intestinal peptide – VIP) secreted by neuroblastoma cells may cause intractable watery diarrhea (6). In our case, although hyperaldosteronism is supposed to cause alkalosis, acidosis was the prominent finding. This acidosis is thought to be caused by intractable watery diarrhea which is related to increased VIP secretion from neuroblastoma cells, and however long-lasting diarrhea may have contributed to deepening of hypokalemia. The spot urine potassium/creatinine ratio less than 1.5 suggests poor intake, gastrointestinal loss or a shift of potassium into the intracellular space (7). In our case, the spot urine potassium/creatinine ratio was found 0.18, and although hyperaldosteronism, the urine fractional excretion of potassium was within normal limits (7.2%). Both ratios support the consideration of severe gastrointestinal potassium loss. The renin-angiotensin-aldosterone system (RAAS) is shown to be overly activated by disorders which cause a drop in renal perfusion pressure and a loss of blood volume (8,9). Nephrotic syndrome is a disorder of the glomeruli that lead to a reduction of plasma volume since there is no plasma albumin to hold the water within the vascular space, and besides, RAAS stimulation is expected due to the decreased volume (8). Usberti *et al.* had investigated sodium retention in 20 nephrotic patients with normal renal functions. Their study suggests that the patients who have normal blood volume are very sensitive to even small changes of blood volume, and respond to volume changes by over activation of RAAS (10). The RAAS is over activated in patients with aortic coarctation in which lower body blood pressure and renal perfusion may be reduced; therefore results in hypertension. Kendirli *et al*. reported a patient with coarctation of the aorta who’s RAAS induced hypertension was aggravated by dehydration and controlled by fluid therapy (9). In addition, neuroblastoma cells were shown to express angiotensin 3 receptor (AT3) which has a role in the regulation of blood flow, although neither renin, angiotensin nor aldosterone are secreted by those cells (11). In our case, AT3 expression by neuroblastoma may have helped with RAAS activation. Moreover, dehydration due to the intractable diarrhea may have contributed to RAAS over stimulation and hyperaldosteronism by reducing intravascular volume, hence the renal blood flow. Vasoactive intestinal polypeptide (VIP) is known to increase catecholamine secretion by stimulating medullary chromaffin cells with paracrine mechanisms, thus are likely to mediate aldosterone secretion following catecholamine release. VIP has been shown to have important role in this subject for the past few years (12-14). In our patient, we think that VIP secreted by neuroblastoma contributed to hyperaldosteronism. Normally, renin is supposed to be suppressed in aldosterone-secreting tumors (3), whereas not suppressed, even higher than normal levels of plasma renin activity in our patient suggest a secondary hyperaldosteronism etiology. In our case, the first two ultrasound scan showed nothing in terms of abdominal mass. Some of abdominal masses can grow up fast in few days and they cannot be examined by palpation during this time. Also ultrasound can have some pitfalls in screening calcified soft-tissues and thus, the mass can be overlooked. It has to be kept in mind that the ultrasound is a subjective interpretation which depends on the experience of the investigator and the condition of the patient at that very moment of investigation. CT scan is recommended to evaluate especially smaller adrenal masses, and should be performed to ensure the diagnosis in suspicious cases like ours.

## Conclusion

Hyperaldosteronism can be presented by unexpected atypical forms as in our patient. To sum up, imaging techniques and pathological diagnosis have to be considered to determine the etiology of hyperaldosteronism.

## Authors’ Contributions

KG wrote the primary draft. AOA reported the pathology. NU reported radiologic findings. UB, STK and FS provided extensive intellectual contribution. KG and EB provided extensive senior intellectual contribution and prepared the final manuscript.

## Conflict of interests

None to declare.

## Ethical considerations

Ethical issues (including plagiarism, data fabrication, double publication) have been completely observed by the author.

## Funding/Support

No financial support by any institution.
